# Construction of Flexible Kaolin/Chitin Composite Aerogels and Their Properties

**DOI:** 10.3390/gels12010076

**Published:** 2026-01-15

**Authors:** Meng He, Yujia Huang, Zhicheng Cui, Ziyue Cheng, Weiwei Cao, Gan Wang, Wei Yao, Mengna Feng

**Affiliations:** 1 School of Materials Science and Engineering, Yancheng Institute of Technology, Yancheng 224051, China; huang13913906481@163.com (Y.H.); 15851145569@163.com (Z.C.); 17849954343@163.com (Z.C.); 18961997828@163.com (G.W.); xiaoniu1981@126.com (W.Y.); fmn0705@ycit.edu.cn (M.F.); 2 Hebei Haien Rubber & Plastic Products Co., Ltd., Cangzhou 061800, China; caoweiwei1982@126.com; 3 Sichuan Province Engineering Technology Research Center of Novel CN Polymeric Materials, Chengdu 611731, China

**Keywords:** chitin aerogels, kaolin, hemostatic dressings

## Abstract

In this work, kaolin/chitin (K/Ch) composite aerogels with different mass ratios were successfully fabricated via a freeze–drying approach. The influence of kaolin content on the microstructure, properties and hemostatic performance of the composite aerogels was systematically investigated. The results demonstrated that the incorporation of kaolin endowed the chitin-based aerogels with tunable porous structures, excellent water absorption capacity (up to 4282% for K_0.25_/Ch_2_), and enhanced water retention (73.7% for K_2_/Ch_2_ at 60 min). Moreover, the K/Ch composite aerogels exhibited good biodegradability, no cytotoxicity (cell viability > 91.9%), and no hemolysis (hemolysis rate < 1.5% at all test concentrations). In vitro hemostatic evaluations revealed that the composite aerogels exhibited rapid blood coagulation (blood clotting time of 16 s for K_2_/Ch_2_) with a blood coagulation index (BCI) as low as 0.5%, which was attributed to the synergistic effect of the physical adsorption of chitin and the coagulation cascade activation by kaolin. These findings indicated that the K/Ch composite aerogels could be used as novel natural hemostatic materials for potential effective and rapid hemostasis.

## 1. Introduction

Blood accounts for approximately 7–8% of the total human body weight, playing a vital role in oxygen transport, nutrient delivery, and immune regulation [1]. Uncontrolled hemorrhage, especially from deep wounds or non-compressible sites (e.g., femoral artery injury caused by traffic accidents), remains a leading cause of death in prehospital emergency and surgical settings [2,3]. Timely and effective hemostasis is therefore critical to improving patient survival rates [4]. Conventional hemostatic materials (e.g., gauze, fibrin glue) show satisfactory efficacy for superficial traumatic bleeding but fail to address the hemostatic demands of deep, penetrating, or incompressible wounds due to their poor absorption capacity, inadequate adhesion, or limited biocompatibility. Ideal hemostatic materials should integrate excellent hemostatic efficiency, biocompatibility, degradability, high porosity, and rapid liquid absorption [5,6]. Aerogels exhibit high porosity, good flexibility and high specific surface area, which allow them to absorb blood rapidly to ultimately achieve rapid hemostasis. Various aerogels have been successfully utilized in the rapid hemostasis application [7,8,9].

Currently, natural polysaccharide-based materials such as chitosan [10], collagen [11], gelatin [12], sodium alginate [13] and cellulose [14], have been extensively explored for hemostatic applications. Chitin, a natural polysaccharide extracted from crustacean shells, is non-toxic, biocompatible, biodegradable and non-irritating, making it a promising matrix for biomedical materials [15,16]. At the same time, a series of modified chitin and its derivatives hemostatic agents have been developed by a combination of other substances, which possess attractive and enhanced properties [17,18]. Chitin-based porous aerogels can rapidly absorb biological fluids via capillary action due to their high porosity and abundant hydrophilic groups (hydroxyl and amide groups) [19,20,21]. However, pure chitin aerogels suffer from insufficient hemostatic activity, limiting their practical application in severe bleeding scenarios. The incorporation of other materials, such as inorganic materials and polymers, could improve the hemostatic activity of chitin [5,22,23].

Kaolin, a well-known inorganic hemostatic agent, exerts its procoagulant effect through two synergistic mechanisms: the physical concentration of platelets and thrombin via rapid water absorption, and the concurrent activation of the intrinsic coagulation pathway by triggering Factor XII [24,25]. This dual action effectively accelerates the initiation and propagation of the clotting cascade, contributing to its reliable hemostatic performance. Additionally, kaolin is free of animal- or human-derived proteins, thus avoiding potential allergic reactions [26]. However, the poor processability and low structural stability of pure kaolin restrict its direct utilization as a hemostatic dressing. It is noted that kaolin is frequently incorporated as a filler to improve the hemostatic activity of various polymers including chitosan, polyurethane, sodium alginate and cellulose acetate, etc. [25,26,27,28]. In their findings, the hemostasis time could be significantly shortened with the incorporation of kaolin.

To overcome the aforementioned limitations of the individual components, combining chitin with kaolin is anticipated to produce synergistic effects. Chitin provides a porous, hydrophilic matrix for rapid liquid absorption and cell compatibility, while kaolin enhances hemostatic efficiency and mechanical stability. In this study, chitin/kaolin (K/Ch) composite aerogels with different mass ratios were fabricated via the processes of blending, crosslinking and freeze–drying. A comprehensive characterization was performed to investigate their morphologies and structure. Physicochemical properties including thermal stability, water absorption, water retention, and water vapor transmission rate (WVTR) were evaluated. Moreover, in vitro biodegradability, cytocompatibility, hemocompatibility, and hemostatic performance (coagulation time and BCI) were systematically assessed. The primary objectives of this work were to clarify the influence of kaolin content on the structure and properties of chitin-based aerogels and give optimal K/Ch ratio for rapid and effective hemostasis, hoping to provide novel chitin-based aerogels for potential wound dressings.

## 2. Results and Discussion

### 2.1. Appearances and Morphologies of the K/Ch Aerogels

**Figure 1:**
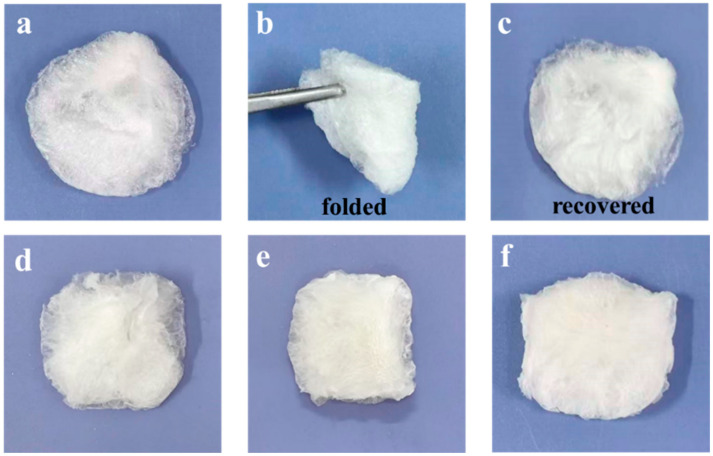
Photographs of the K_0.25_/Ch_2_ aerogel at original (**a**), folded (**b**) and recovered (**c**) states, and photographs of K_0.5_/Ch_2_ (**d**), K_1_/Ch_2_ (**e**)and K_2_/Ch_2_ (**f**) aerogels.

As shown in Figure 1, a series of K/Ch composite aerogels with different kaolin contents were successfully prepared. The K_0.25_/Ch_2_ aerogel had relatively loose edge and showed a white color, which could recover its original shape after folding, indicating good flexibility. With the increase in kaolin content, the K_0.5_/Ch_2_, K_1_/Ch_2_ and K_2_/Ch_2_ aerogels showed light-yellow color and the edges of the sponges became denser. All the aerogels were intact and exhibited homogenous morphology, indicating good miscibility between chitin and kaolin.

**Figure 2:**
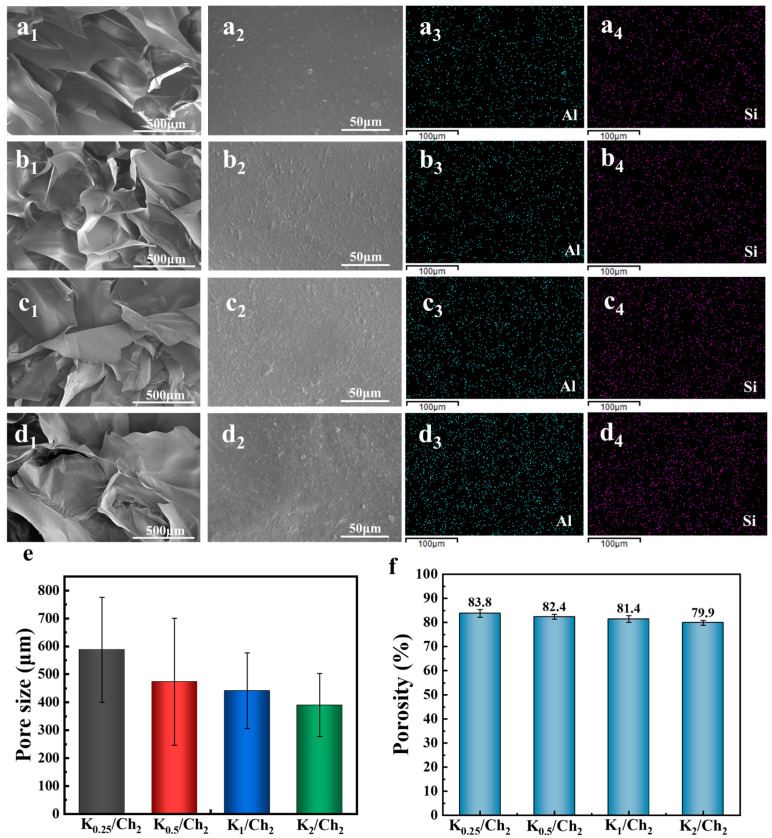
SEM images (**a_1_**–**d_1_**,**a_2_**–**d_2_**) and elements mappings (**a_3_**–**d_3_** for Al and **a_4_**–**d_4_** for Si) for the K_0. 25_/Ch_2_ (**a**), K_0. 5_/Ch_2_ (**b**), K_1_/Ch_2_ (**c**) and K_2_/Ch_2_ (**d**) aerogels, respectively, and the corresponding pore size distribution (**e**) and porosity (**f**).

SEM was used to evaluate the microstructure change in the K/Ch aerogels varying with the kaolin content. All the K/Ch composite aerogels exhibited porous structures (Figure 2(a_1_–d_1_)), and the average pore size decreased from 588.5 μm for K_0.25_/Ch_2_ to 389.9 μm for K_2_/Ch_2_ with increasing kaolin content (Figure 2e). This trend could be primarily attributed to the physical filling effect of the kaolin particles within the chitin matrix. During the sol–gel and subsequent freeze–drying processes, the dispersed kaolin particles acted as fillers that occupied space and interfered with the growth of ice crystals, which served as templates for the macropores [29,30]. Consequently, a higher kaolin content resulted in more significant space occupation and obstruction, leading to the formation of smaller average pores in the final aerogel architecture. Porosity is also one of the basic parameters of aerogels for their potential as wound dressings. It is noted that the porosity of K_0.25_/Ch_2_ decreased from 83.8 to 82.4, 81.4 and 79.9% for K_0.5_/Ch_2_, K_1_/Ch_2_ and K_2_/Ch_2_, respectively, which was possibly due to the above physical filling effect [29,30]. There were many particles on the chitin pore walls (Figure 2(a_2_–d_2_)), which were confirmed to be kaolin by the results of elements mappings of Al and Si (Figure 2(a_3_–d_3_,a_4_–d_4_)), indicating the successful incorporation of kaolin in the chitin matrix.

### 2.2. Structure of the K/Ch Aerogels

**Figure 3:**
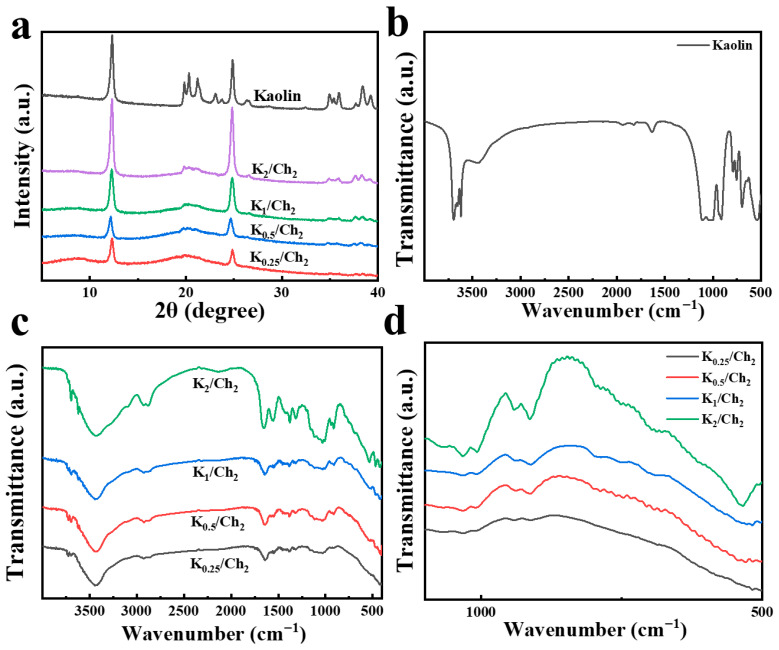
XRD patterns of kaolin and the K_0.25_/Ch_2_, K_0.5_/Ch_2_, K_1_/Ch_2_ and K_2_/Ch_2_ composite aerogels (**a**). FTIR spectra of kaolin (**b**), and the K_0.25_/Ch_2_, K_0.5_/Ch_2_, K_1_/Ch_2_ and K_2_/Ch_2_ composite aerogels (**c**,**d**). d depicts the local magnified spectra of c.

The XRD analysis of the K/Ch composite aerogels (Figure 3a) revealed characteristic peaks at 2θ~8.0° and 20.0°, which are assigned to the (020) and (110) planes of chitin [31]. According to the previous work, ECH disrupts the ordered packing of chitin chains in the crystalline regions, leading to a decrease in overall crystallinity, which is directly reflected in the XRD patterns as weakened and broadened peaks [31,32,33]. Notably, these above two peaks were weak and became weaker with the increase in kaolin content due to the ECH chemical crosslinking effect and interaction with kaolin [33]. The peaks at 2θ = 12.3°, 19.8°, 24.8°, 34.9°, and 37.6° corresponded to the (001), (100), (002), (110), and (003) crystal planes of kaolin, respectively. Two main characteristic peaks at 2θ~12.3° and 24.7° appeared in the K/Ch composite aerogels, whose intensity increased gradually with increasing the kaolin content, further confirming the incorporation of kaolin in the aerogels.

Figure 3b–d show the FTIR spectra of the kaolin and K/Ch composite aerogels. The absorption peaks at around 3438 cm^−1^ and 2878 cm^−1^ for K_0.25_/Ch_2_ were assigned to the -OH and -CH stretching vibrations, respectively. The peaks at around 1643 cm^−1^, 1553 cm^−1^ and 1376 cm^−1^ were attributed to the vibrations of amide I (ν_C=O_), amide II (δ_N-H_) and amide III (ν_C-N_), respectively, which corresponded to the main structure of chitin. As for the composite aerogels, the peak at 3705 cm^−1^ corresponded to the -OH stretching vibration of kaolin (Figure 3c,d). The peaks at 1034 and 469 cm^−1^ were attributed to the stretching and bending vibrations of the Si–O bonds, respectively, while the peak at 541 cm^−1^ was associated with the Si–O–Al bending vibration (Figure 3b–d) [30,31,34]. Meanwhile, the characteristic peak intensity of kaolin increased gradually in the K/Ch composite aerogels with increasing the kaolin content (Figure 3c,d), further confirming the existence of kaolin. It is noted that the -OH stretching vibration peak for K_0.25_/Ch_2_ shifted gradually to a lower wavenumber at 3432 cm^−1^ for K_2_/Ch_2_, indicating the existence of hydrogen bonding interaction between chitin and kaolin.

### 2.3. Properties of the K/Ch Aerogels

**Figure 4:**
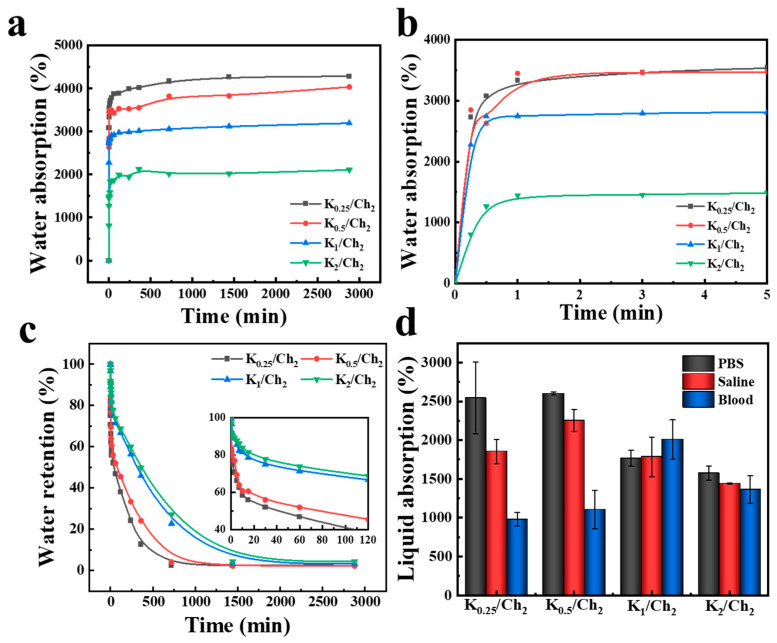
Water absorption dynamic curves (**a**,**b**), water retention curves (**c**), and liquid absorption ratios of the K/Ch composite aerogels (**d**).

Materials with high absorption capacity can promote hemostasis by rapidly absorbing water in blood to concentrate coagulation components, thereby helping reduce casualties caused by excessive blood loss [35]. Figure 4a,b show the water absorption kinetics curve of the K/Ch aerogels. The water absorption rates of K_0.25_/Ch_2_, K_0.5_/Ch_2_, K_1_/Ch_2_ and K_2_/Ch_2_ reached 4282, 4036, 3194 and 2107%, respectively, which could satisfy the water absorption capacity requirement of dressings due to the good hydrophilicity nature of chitin. The gradual decrease in water absorption rate with increasing kaolin content was due to two main factors. First, the porosity and pore size of the aerogels reduced as kaolin content increased (Figure 2e,f), leading to a decrease in their water storage capacity. Second, kaolin enhanced the rigidity of the aerogel pore walls, making the aerogels less prone to deformation and swelling upon water absorption, thereby further contributing to the decrease in their water absorption rate.

The water retention capacity of the K/Ch composite aerogels was quantitatively assessed. As shown in Figure 4c, all composite aerogels demonstrated effective water-holding properties. The 60 min water retention rates increased significantly from 46.8% to 71.4% as the kaolin content increased from 0.25 wt% to 1 wt% (for K_0.25_/Ch_2_ to K_1_/Ch_2_). However, a further increase to 2 wt% (K_2_/Ch_2_) yielded only a marginal improvement to 73.7%, indicating that ~1 wt% represents an optimal content for maximizing retention within this timeframe. This positive correlation was maintained over longer durations. After 240 min, the retention rates increased gradually from 24.1% for K_0.25_/Ch_2_ to 59.9% K_2_/Ch_2_. The enhancement in water retention was attributed to a synergistic interplay of microstructural and physicochemical modifications by kaolin. First, on a molecular level, the surface -OH groups on kaolin could form strong hydrogen bonding interaction with the functional groups (-OH, -NH_2_ and -NHCOCH_3_) of chitin, which could provide a direct mechanical anchorage. The anchored, rigid kaolin particles functioned as reinforcements within the chitin matrix (Figure 2(a_2_–d_2_)), which could inhibit the collapse and shrinkage of pores during dehydration, thereby preserving a more stable and porous network that to trap water physically. Second, the surface -OH on kaolin and hydrophilic groups of chitin provided sites for forming hydrogen bonds with water molecules, which could bind water and increase the energy required for water evaporation. Crucially, these two effects are interdependent and mutually reinforcing. The kaolin-reinforced, mechanically robust microstructure maintained the architectural integrity and high surface area of the pores, which in turn ensured sustained access to the hydrophilic binding sites. Concurrently, the water molecules bound to these sites helped to maintain a humid internal microenvironment, which could reduce capillary forces that drive structural shrinkage, ultimately leading to the superior water-holding capacity of the kaolin–chitin composites.

Figure 4d depicts PBS, Saline and Blood uptake capacity of all the K/Ch aerogels. Notably, composite aerogels demonstrated high absorption capacity, with minimum ratios of 1568.9%, 1440.3% and 979.8% for PBS, Saline and Blood, respectively, indicating good liquid absorption properties and showing potentials as wound dressings for biomedical applications. Three-dimensional hydrophilic aerogel network (Figure 2(a_1_–d_1_)) allowed the penetration of the above liquids through capillary action, endowing the composite aerogels with good liquid absorption properties. Variations in absorption among different liquids for different aerogels were possibly due to the differences in their micro-structure and component contents.

**Figure 5:**
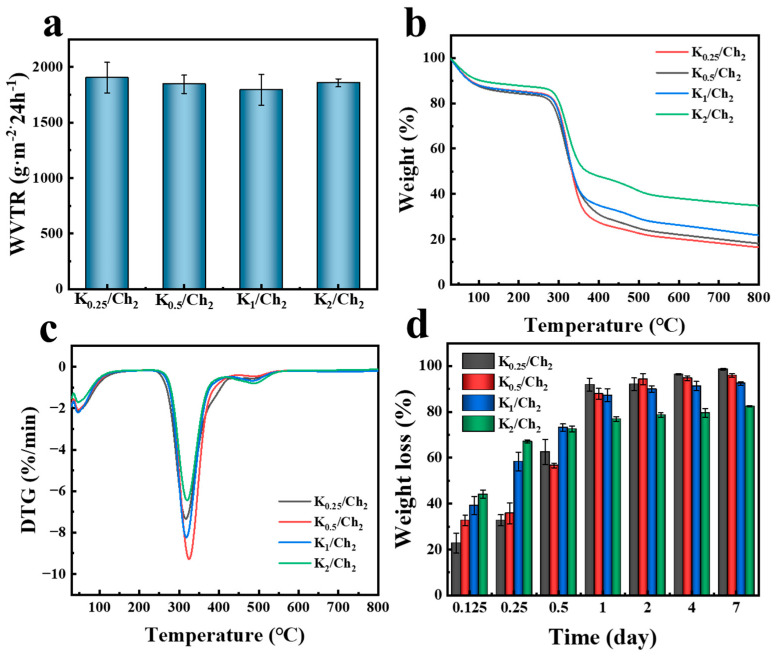
WVTR (**a**), TG and DTG curves (**b**,**c**), and in vitro degradation rate (**d**) of the K/Ch aerogels.

A suitable WVTR is essential for wound dressings to maintain a healing-promoting moist environment, preventing excessive drying or accumulation of exudate. Figure 5a shows the WVTR results of all the composite aerogels. The K_0.25_/Ch_2_ aerogel, with the lowest kaolin loading, exhibited the highest WVTR value of 1903.2 g/m^2^/d. As the kaolin content increased, the WVTR values changed to 1845.9, 1793.6, and 1858.6 g/m^2^/d for K_0.5_/Ch_2_, K_1_/Ch_2_ and K_2_/Ch_2_, respectively. This subtle reduction in WVTR could be primarily attributed to the kaolin microstructural modification. As we mentioned above, the rigid kaolin particles partially occupied or constricted the pores within the chitin matrix, leading to increased tortuosity of the vapor diffusion pathways. All the composite aerogels exhibited WVTR values that significantly exceeded the average water vapor evaporation rate of human skin, a feature that is crucial for effective wound management by reducing the risk of maceration and facilitating healing [36,37].

Thermal stability is also important for medical dressings, which usually need to undergo a high-temperature autoclave process. Figure 5b,c show the TG and DTG curves of the K/Ch composite aerogels. The TGA curves of all samples exhibited a minor weight loss below 100 °C, attributable to the evaporation of absorbed moisture. No obvious decomposition event occurs below 200 °C, which corresponded to the thermal degradation of the chitin polymer backbone [38]. Notably, the maximum decomposition temperature (T_max_) of all K/Ch composite aerogels exceeded 300 °C, which was caused by the thermal cleavage of the β-(1,4)-glycosidic bonds (C-O-C) and deacetylation of chitin [39,40]. These results confirmed the inherent thermal stability of all the K/Ch aerogels and potential feasibility for high-temperature sterilization processes, which are common to medical device preparation. Beyond the primary decomposition, the aerogels showed a more gradual weight loss at 350–800 °C [41]. Moreover, the residual weight values at 800 °C for the K/Ch aerogels were consisted with the reported work [42,43,44], which increased with increasing the content of kaolin, further confirming the incorporation of kaolin.

The in vitro degradation profiles of the K/Ch aerogels were assessed in PBS containing 0.45 mg/mL lysozyme (Figure 5d). All aerogels exhibited a rapid initial degradation phase. Within 3 h (0.125 d), each sample underwent a mass loss of above 30%, which further increased to over 55% at 6 h (0.25 d). Notably, the degradation rate of the composite aerogels generally decreased with increasing kaolin content, which could be attributed to two primary mechanisms associated with the incorporated kaolin [45]. First, the dispersed kaolin particles acted as a physical barrier within the chitin matrix (Figure 2(a_2_–d_2_)), impeding the penetration and diffusion of lysozyme enzymes to their cleavage sites on the polymer chains. Second, interactions such as between the surface hydroxyl groups of kaolin and the functional groups (e.g., -OH, -NHCOCH_3_) of chitin could reduce polymer chain mobility and accessibility, making them less susceptible to enzymatic hydrolysis. The weight loss values for all the aerogels were above 82.5% at 7 d, indicating good biodegradability and their potential as biodegradable wound dressings.

### 2.4. Biological Evaluation of the K/Ch Aerogels

**Figure 6:**
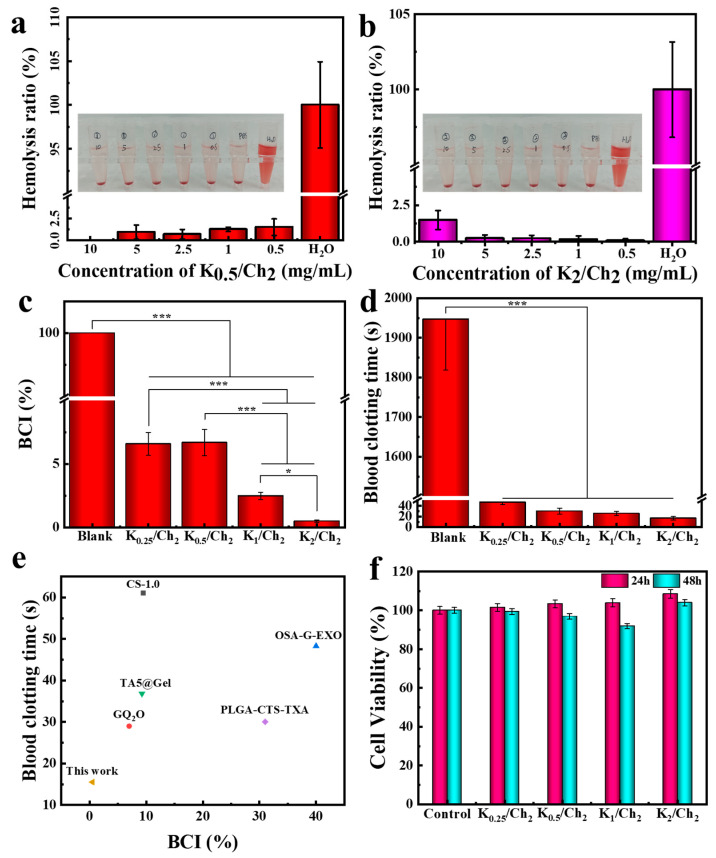
The hemocompatibility of K_0.5_/Ch_2_ (**a**) and K_2_/Ch_2_ (**b**), BCI (**c**) and the blood clotting time (**d**) of all the K/Ch aerogels, BCI and blood clotting time values of K_2_/Ch_2_ compared with other recently reported sponges (GQ_2_O [49], TA5@Gel [50], CS-1.0 [51], PLGA-CTS-TXA [52], OSA-G-EXO [53]) (**e**), and results of the cytotoxicity tests of all the K/Ch aerogels (**f**). A *p*-value of <0.05 was defined as statistically significant and denoted by (*), and *p* < 0.001 as highly significant (***).

The hemocompatibility of hemostatic materials is critically evaluated by their hemolysis rate, which reflects the tendency to damage erythrocytes [33]. As shown in Figure 6a,b, both K_0.5_/Ch_2_ and K_2_/Ch_2_ exhibited hemolysis rates below 1.5% across all tested concentrations (0.5, 1, 2.5, 5, and 10 mg/mL). These values were much less than the 5% threshold generally accepted for biomedical materials, indicating excellent erythrocyte compatibility and satisfying the requirement for hemolytic safety as dressings.

BCI was employed to quantitatively evaluate the hemostatic potency of the K/Ch aerogels in vitro. A lower BCI signifies a more rapid and effective clotting process. As shown in Figure 6c, the BCI values of K_0.25_/Ch_2_, K_0.5_/Ch_2_, K_1_/Ch_2_ and K_2_/Ch_2_ were determined to be 7.0%, 6.2%, 2.4%, and 0.5%, respectively. This progressive decrease in BCI with higher kaolin loading demonstrated a clear concentration-dependent enhancement of coagulation efficiency. This trend was directly interpreted as a consequence of the increased density of negatively charged surfaces available for contact activation. As we mention above, kaolin is known to specifically adsorb and induce the auto-activation of coagulation Factor XII (FXII, Hageman factor) [24,25,46,47]. The activation of FXII is the critical first step of the intrinsic coagulation pathway, leading to a cascade of proteolytic reactions that culminate in fibrin clot formation [48]. Therefore, a higher kaolin content within the K/Ch aerogel architecture provided a greater number of activating sites, leading to more efficient and rapid initiation of this coagulation cascade, which was reflected in the successively lower BCI values. Notably, K_2_/Ch_2_ exhibited significantly lower BCI compared to many reported hemostatic sponges [49,50,51,52,53] (Figure 6e), underscoring their excellent coagulation performance.

The in vitro blood clotting time was also measured to directly assess the hemostatic capability of the K/Ch composite aerogels. As presented in Figure 6d, all the K/Ch aerogels induced rapid coagulation within 50 s. In contrast, no clot formation was observed in the control group after 30 min. The clotting time for K_0.25_/Ch_2_ decreased gradually from 46 s to 32 s, 24 s, and 16 s for K_0.5_/Ch_2_, K_1_/Ch_2_ and K_2_/Ch_2_. This inverse correlation between clotting time and kaolin content reinforced the above interpretation that the hemostatic capability was concentration-dependent, governed by the availability of FXII-activating surfaces. Compared with other reported sponges [49,50,51,52,53], the extremely short time of 16 s for K_2_/Ch_2_ confirmed a superior hemostatic efficacy of the composite aerogels due to a synergistic hemostatic mechanism. Apart from the above effect of kaolin, the cationic nature of the chitin surface contributed to the clumping of red blood cells and platelets, leading to the formation of an initial cellular plug. Furthermore, the highly porous aerogel structure facilitated rapid plasma absorption and the concentration of coagulation components, thereby accelerating the coagulation cascade. Therefore, the increasing kaolin content in K/Ch aerogels directly enhanced hemostatic performance by providing more sites for contact activation of the intrinsic coagulation pathway. This effect, synergized with the cellular-aggregating property of chitin and the plasma-absorbing porous structure, results in the observed concentration-dependent decrease in both BCI and clotting time, culminating in the exceptionally rapid hemorrhage control of K_2_/Ch_2_.

The cytocompatibility of the K/Ch aerogels was assessed using the MTT assay after incubating L929 fibroblasts with the extracts of composite aerogel. As shown in Figure 6f, all the K/Ch aerogels exhibited cell viability values of above 101.5% at 24 h and 91.9% at 48 h, meeting the requirements for non-cytotoxicity according to ISO 10993-5 [54]. Notably, the viability values above 100% at 24 h indicated a mild proliferative effect of the aerogel extracts on L929 cells. This observed cell-promoting tendency was due to two main factors. First, kaolin can gradually release silicon ions in the physiological media. Silicon at appropriate concentrations has been reported to support metabolic activity and proliferation in certain cell types [55,56]. Second, the incorporation of kaolin into the chitin matrix increased surface roughness of the aerogel pore walls (Figure 2(b_1_–b_4_)), which could enhance protein adsorption and provide more anchoring sites for cell adhesion and subsequent growth.

## 3. Conclusions

In this study, a series of K/Ch composite aerogels with different mass ratios were successfully fabricated via a blending–crosslinking–freeze–drying approach. The microstructure, physicochemical properties, and hemostatic performance of the composite aerogels were systematically investigated. The incorporation of kaolin endowed the composite aerogels with tunable porous structures, excellent comprehensive properties—including a maximum water absorption capacity of 4282% (K_0.25_/Ch_2_), enhanced water retention (73.7% for K_2_/Ch_2_ at 60 min), good thermal stability (T_max_ > 300 °C) and prominent biodegradability (over 82.5% mass loss within 7 days). K/Ch also exhibited non-cytotoxicity (cell viability > 91.9% after 48 h) and no hemolysis (hemolysis rate < 1.5% across all test concentrations). Notably, the hemostatic performance was significantly improved with increasing kaolin content, and the K_2_/Ch_2_ aerogel achieved an ultra-low BCI of 0.5% and a rapid blood clotting time of 16 s, attributed to the synergistic effect of chitin’s promotion of erythrocyte and platelet aggregation and kaolin’s activation of the intrinsic coagulation cascade. Collectively, these K/Ch composite aerogels integrated multiple advantages required for hemostatic dressings, with K_2_/Ch_2_ showing the most prominent comprehensive performance, making it a promising natural candidate as wound dressings.

## 4. Materials and Methods

### 4.1. Materials

Raw chitin powder was provided by Golden-Shell Biochemical Co., Ltd. (Yuhuan, China). Kaolin (particle size (D50) ~ 1.92 μm, purity ≥ 96%) was purchased from Guzhang County Shanlin Shiyu Mineral Products Co., Ltd. (Xiangxi, China). NaOH, urea were obtained from Sinopharm Chemical Reagent Co., Ltd. (Shanghai, China). Epichlorohydrin (ECH) was purchased from Shanghai Aladdin Bio-Chem technology Co., Ltd. (Shanghai, China). Lysozyme was procured commercially from Hefei Bomei Biotechnology Co., Ltd. (Hefei, China). Anticoagulated porcine whole blood was provided by Zhengzhou Pingrui Biotechnology Co., Ltd. (Zhengzhou, China).

### 4.2. Preparation Process of the K/Ch Aerogels

NaOH and urea were dissolved in distilled water and treated using an ultrasonic cleaner (KQ5200E, Kun Shan Ultrasonic Instruments Co., Ltd., Suzhou, China) for 3–5 min to form a homogeneous solution. Kaolin was added to the above solution, followed by ultrasonic dispersion to ensure uniform distribution of kaolin particles. Desired amounts of chitin were added to the above kaolin/alkali/urea solutions, which were ultrasonicated for 3–5 min. The resultant mixtures were then frozen for 4 h, taken out, stirred gently when surface ice crystals appeared, and frozen again for another 4 h. This freeze–thaw cycle was repeated 3 times to achieve 3 wt% chitin solution. ECH was slowly added to the chitin/kaolin solutions with mechanical stirring for 15 min at room temperature. The crosslinked solutions were centrifuged at 5000 rpm for 10 min to remove air bubbles, then poured into cylindrical molds and left to stand at room temperature for 24 h for molding. The molded chitin/kaolin composite hydrogels were soaked in distilled water until the pH reached neutral. The neutral chitin hydrogels were wrapped in tin foil, frozen for 24 h, and then freeze-dried for 48 h using a freeze dryer (LGJ-12, Beijing Songyuan Huaxing Technology Develop Co., Ltd., Beijing, China). The resulting composite aerogels were named K_0.25_/Ch_2_, K_0. 5_/Ch_2_, K_1_/Ch_2_ and K_2_/Ch_2_ according to the kaolin mass fractions of 0.25 wt%, 0.5 wt%, 1 wt%, and 2 wt%, respectively.

### 4.3. Characterization

The cross-sectional morphologies of the K/Ch aerogels were examined using a field emission scanning electron microscope (FE-SEM; Nova NanoSEM 450, FEI, Tokyo, Japan). Before imaging, the composite aerogels were immersed in liquid nitrogen for rapid freezing, manually fractured, and then freeze-dried. The exposed cross-sections of the dried specimens were coated with a thin gold layer via sputtering to enhance conductivity during SEM observation. The average pore size for different aerogels was calculated by measuring 20 pores of each sample using Nano Measurer 1.2 (Fudan University, Shanghai, China). XRD measurements were carried out on an X’Pert3 Powder diffractometer (PANalytical, Almelo, Holland) equipped with Cu Kα radiation. The diffraction patterns were recorded over a 2θ range of 5° to 40°. Prior to analysis, all K/Ch aerogels were ground into powder, transferred into designated plastic containers, and dried under vacuum for 48 h. FTIR spectroscopy was performed on a NEXUS-670 spectrometer (Nicolet, Madison, WI, USA) spanning the wavenumber region of 400–4000 cm^−1^. For FTIR measurement, each powdered aerogel sample was thoroughly mixed with spectroscopic-grade KBr and pressed into pellets under a load of 10 tons for 2 min. Thermogravimetric analysis (TGA) was conducted on a TGA5500 instrument (TA Instruments, New Castle, DE, USA) to evaluate the thermal stability of the K/Ch aerogels. Samples were heated from 30 °C to 800 °C at a constant rate of 10 °C/min under a controlled atmosphere. Derivative thermogravimetry (DTG) curves were also derived from the TGA data.

In the water absorption assay, the K/Ch aerogels were first dried at 60 °C for 1 d and weighed as W_0_. Subsequently, the samples were immersed in distilled water for a designated duration. Following immersion, any residual surface water was carefully blotted off with filter paper before the aerogels were weighed again (W_t_). The water absorption ratios were then determined using Equation (1).

(1) $$\mathrm{Water}\;\mathrm{absorption}\;\mathrm{ratio}\;\left(\%\right)=\frac{W_{t}\;-\;W_{0}}{W_{0}}\;\times \;100\%\;$$

To evaluate liquid absorption, the dried K/Ch aerogels (initial weight W_0_) were soaked in physiological saline (PS), anticoagulated porcine whole blood (Blood) and phosphate-buffered solution (PBS), respectively. Following a 24 h soaking, the aerogel samples were taken out, blotted with filter paper to remove excess liquid, and then weighed to obtain the wet weight (W_1_). The absorption ratio for each medium was subsequently calculated using Equation (2).

(2) $$\mathrm{Absorption}\;\mathrm{ratio}=\frac{W_{1}\;-\;W_{0}}{W_{0}}\;\times \;100\%\;$$

The water retention test began by fully hydrating the completely dried aerogels (M_0_) in distilled water. After removing excess surface water, the saturated samples were weighed (M_s_). They were then stored at room temperature, and their weight (M_t_) was recorded at designated intervals during the subsequent evaporation. The retained water content was calculated using Equation (3).

(3) $$\mathrm{Water}\;\mathrm{retention}\;\mathrm{ratio}\;\left(\%\right)=\frac{M_{t}\;{-\;M}_{0}}{M_{s}\;-\;M_{0}}\;\times \;100\%\;$$

Porosity was determined via the liquid displacement method using ethanol (density ρ = 0.785 g/cm^3^). For porosity assessment, the aerogels were first sectioned into uniform cylinders measuring approximately1 cm in diameter and 0.5 cm in height. The dry weight (w_2_) of each cylinder was recorded. Subsequently, the samples were fully immersed in ethanol at ambient temperature for 60 min to ensure complete pore infiltration. After removal from the solvent, any residual ethanol on the external surfaces was carefully blotted away. The samples were then immediately weighed again to obtain the wet mass (w_1_). The porosity of the aerogels was calculated using Equation (4):

(4) $$\mathrm{Porosity}\;\left(\%\right)\;=\;\frac{w_{1}\;-{\;w}_{2}}{\rho V}\;\times \;100\%\;$$

where V is the volume of the cylindrical specimen.

The aerogels (1 cm × 1 cm) were thoroughly dried and weighted (m_0_). After sterilization, the samples were incubated in PBS containing lysozyme (1 mg/mL) at 37 °C with 80 rpm agitation, with the PBS refreshed every three days. At designated time points (0.125, 0.25, 0.5, 1, 2, 4, and 7 days), composite aerogels were collected and weighed (m_t_) after complete drying. The degradation rates of aerogels were then calculated using Equation (5):

(5) $$\mathrm{In}\;\mathrm{vitro}\;\mathrm{degradation}\;\mathrm{rate}\;=\;\frac{m_{0}\;-{\;m}_{t}}{m_{0}}\;\times \;100\%\;$$

To mimic the moist conditions of an exudative wound bed, the water vapor transmission rate (WVTR) of K/Ch aerogels was evaluated. Each sample was used to seal the opening of a glass bottle containing ultrapure water, maintaining an air gap of less than 1 mm between the water surface and the material. The assemblies were then placed in sealed chambers under controlled conditions (37 °C and 75% relative humidity, maintained using saturated NaCl solutions). A bottle with no aerogel served as the blank reference. Mass changes in water in the cup were recorded to quantify water vapor transmission after 1 d. The WVTR (g/m^2^/d) was calculated according to Equation (6).

(6) $$\mathrm{WVTR}=\frac{W_{1}-W_{0}}{A\;\times \;t}$$

where A is the effective area of the cup opening (m^2^), t is the test time (d), W_0_ is the initial mass of water (g), and W_1_ is the mass of water after 24 h (g).

Cell viability assay (MTT): A selected cell line (L929 fibroblasts, QS-M020, keycell) was recovered from cryopreservation and immediately placed in a constant-temperature water bath cooker (BK-3D). The cryovials were gently shaken to thaw the cryopreservative medium. Once thawed, the cells were diluted by adding them to 5 mL of fresh medium in a sterile centrifuge tube. The cells were collected via centrifugation (1000 rpm, 5 min, room temperature) on a high-speed refrigerated centrifuge (Legend Micro 17R, Thermo, Osterode am Harz, Germany), and the supernatant was discarded. A complete medium formulation containing 10% fetal bovine serum (FBS) (FSP500, ExCell Bio, Suzhou, China) was used to resuspend the cells, which were then seeded into Petri dishes, gently mixed via pipetting, and cultured at 37 °C under 5% CO_2_ and saturated humidity in a CO_2_ constant temperature incubator (NU-5800, NUAIRE, Plymouth, MA, USA). Log-phase cells with good viability were harvested. Cells were seeded at a density of 4 × 10^3^ cells per well in a 96-well plate in 100 µL of complete medium. Incubate overnight at 37 °C, 5% CO_2_ to allow adherence. After 24 h, the culture medium in each well was discarded, the wells were rinsed twice with PBS, and sample extracts diluted to each working concentration were added. The cells were incubated for an additional 24 h or 48 h, followed by the assay. The MTT/CCK8 kit (C0009S and C0037, Shanghai Beyotime Biotechnology Co., Inc., Shanghai, China) was thawed at room temperature; 10 μL of MTT/CCK8 chromogenic solution was added to each well, and the plate was incubated in a cell incubator for 4 h. 100 μL formazan dissolution solution was added to each well and mixed gently. Incubation was continued in the cell incubator to ensure complete formazan dissolution. The absorbance at 450 nm was measured using a microplate reader (SpectraMax Paradigm, Molecular Devices, San Jose, CA, USA), and cell viability was calculated using Equation (7):

(7) $$\mathrm{Cell}\;\mathrm{viability}\;\left(\%\right)\;=\;\frac{A_{\mathrm{test}}}{A_{\mathrm{control}}}\;\times \;100\%\;$$

where A_test_ and A_control_ are the absorption values of the test and control groups, respectively.

Hemolysis test: A 2% (*v*/*v*) red blood cell (RBC) suspension was prepared from defibrinated rat blood (Beijing Vital River Laboratory Animal Technology Co., Ltd., Beijing, China). Briefly, 2 mL of whole blood was mixed with 200 μL of anticoagulant and stirred to remove fibrin. The defibrinated blood was washed with phosphate-buffered saline (PBS) via centrifugation (1000–1500 rpm, 15 min) until the supernatant was clear. The packed RBCs were then resuspended in PBS to a 2% concentration. K/Ch samples at various concentrations (0.5, 1, 2.5, 5, 10 mg/mL) were incubated with the RBC suspension at 37 °C for 1 h. Negative and positive controls were PBS and distilled water, respectively. After incubation, the mixtures were centrifuged, and the absorbance (A) of the supernatant was measured at 540 nm. The hemolysis rate was calculated using Equation (8):

(8) $$\mathrm{Hemolysis}\;\mathrm{rate}\;\left(\%\right)\;=\;\frac{A_{S}\;{-\;A}_{n}}{A_{p}{\;-\;A}_{n}}\;\times \;100\%$$

where A_s_, A_n_ and A_p_ are the absorbance of the sample, negative control, and positive control, respectively.

The K/Ch aerogels (10 mg each) were placed into centrifuge tubes, followed by the addition of 100 μL of anticoagulated porcine whole blood and 20 μL of 0.2 mol/L CaCl_2_ solution in sequence. Timing was initiated immediately after the addition of CaCl_2_. The tubes were tilted to 90° every 3–5 s to observe the flowability of the blood. The clotting time was recorded when no liquid flow was observed upon tilting. A blank control (without any hemostatic agent) was prepared in parallel. The hemostatic potential of each of the K/Ch aerogels was evaluated through whole blood coagulation experiments. For the blood interaction assay, cylindrical aerogel samples were positioned in a culture dish maintained at 37 °C. A mixture of 100 μL of whole blood (containing 3.8 wt% sodium citrate anticoagulant at a blood-to-anticoagulant ratio of 9:1) and 20 μL of 0.2 mol/L CaCl_2_ solution was administered onto each aerogel. Following incubation at 37 °C for 5 min, each sample was transferred into 25 mL of distilled water. The mixture was gently shaken and then allowed to stand for an additional 5 min. Subsequently, the absorbance of the supernatant was determined at 545 nm using an UV spectrophotometer (Mapada, UV-6, Shanghai, China). A blank control was prepared by directly adding 200 μL of whole blood into 25 mL of distilled water. The blood coagulation index (BCI) was calculated using Equation (9):

(9) $$\mathrm{BCI}\;\left(\%\right)\;=\;\frac{{\mathrm{OD}}_{\mathrm{aerogel}}}{{\mathrm{OD}}_{\mathrm{control}}}\;\times \;100\%\;$$

where OD_aerogel_ and OD_control_ depict the absorbance values of the aerogels, and the control group, respectively.

For all statistical analyses (e.g., MTT assay, blood clotting time, BCI) of the K/Ch aerogels, a *p*-value of <0.05 was defined as statistically significant and denoted by (*), *p* < 0.01 as moderately significant (**), and *p* < 0.001 as highly significant (***).

## Data Availability

The date that support the findings of this study are available from the corresponding author upon reasonable request.
